# Using Corpus Linguistics to Investigate Approaches to Oyster Fishery Management Across Political Boundaries

**DOI:** 10.1007/s00267-025-02163-9

**Published:** 2025-04-18

**Authors:** Shannon Fitzsimmons-Doolan, Jennifer Beseres Pollack

**Affiliations:** 1 English Department, Texas A&M—Corpus Christi, Corpus Christi, TX USA; 2 Harte Research Institute, Texas A&M—Corpus Christi, Corpus Christi, TX USA

**Keywords:** Gulf of Mexico, Oyster fisheries, Coastal management, Corpus linguistics, Keyword analysis

## Abstract

Oysters are critical resources that filter water, generate habitat, and safeguard shorelines in coastal and marine ecosystems. Balancing conservation needs with sustainable oyster fisheries is essential for maintaining oyster health and stocks. In the U.S. Gulf of Mexico, oyster resources are managed by five states (Texas, Louisiana, Mississippi, Alabama, and Florida), each with unique approaches and priorities. This study analyzes the most current oyster management guidance document for each state using corpus linguistics techniques—including keyword and concordance analyses—to identify linguistic distinctions that reflect state-specific management priorities. Findings reveal that Florida’s document is the most distinctive, emphasizing oyster stressors and habitat. Louisiana’s document reflects its role as a major oyster producer. Mississippi’s document uniquely highlighted aquaculture as a strategy for recovering from environmental stressors. The theme of oyster restoration is robust in Alabama’s 2021 document but absent in Texas’s 1988 document, highlighting temporal differences in management priorities. In addition, common themes such as state-specific oyster stressors emerged among the distinctions. These results demonstrate how management priorities vary across political boundaries and provide insights for improving regional coordination. This approach offers a framework that can inform natural resource management strategies in other contexts and in other regions globally.

## Introduction

Populations of native oysters are rapidly declining globally due to multiple factors including reduced freshwater delivery from rivers, unsustainable harvests, and disease, affecting provision of ecosystem benefits (Beck et al. 2011; zu Ermgassen et al. 2012; Giglio et al. 2024). Management of oyster resources—particularly in terms of balancing conservation needs with sustainable oyster fisheries—can greatly affect oyster health and stocks (Powell et al. 2012; 2018; McAfee and Connell 2020). However, due to their broad geographical distribution, management of oyster resources can occur as a mosaic of differing practices across political boundaries (Kennedy and Breisch 1983; Nevins et al. 2014; Tracy et al. 2023).

Management of oyster resources is guided by a range of approaches, each shaped by location-specific variables and nested structures of governance (Botsford et al. 1997; Bennett and Satterfield 2018). This continuum of management activities includes restoration efforts, conservation strategies, and ongoing monitoring, which collectively address challenges related to sustainable ecosystems and uses (Ewel 2001; Beck et al. 2011; Kennish 2022). Key approaches to oyster resource management include (1) modeling ecological and harvesting variables to achieve sustainability (e.g., Powell et al. 2012), (2) fisheries management science to guide harvest activities (e.g., Stephenson and Lane 2010), (3) the ecosystem approach, which considers the broader ecological context of oyster habitats (e.g., Freitag et al. 2018), (4) collaborative management that integrates local knowledge and stakeholder participation (e.g., Brown et al. 2021), (5) adaptive planning that allows for flexibility in response to environmental changes (e.g., Pine et al. 2022), and (6) spatial planning that focuses on mapping and monitoring to inform management (e.g., Swam et al. 2022).

Various documents play a critical role in guiding resource management efforts, ranging from formal fisheries management plans that are typically created by state and federal agencies and establish structured management frameworks (Stephenson and Lane 2010), to assessment reports, to management guidance documents that may be produced collaboratively to guide management decisions in situations where no formal plans exist (Pomeroy and Rivera-Guieb, 2005; Gutiérrez et al. 2011; Brown et al. 2021). Sustainable management of natural resources across politically disparate but ecologically connected systems presents significant challenges, particularly when balancing ecological considerations with varying human activities and priorities (Botsford et al. 1977; La Peyre et al. 2022; Scyphers et al. 2014; Kennish 2022). The diversity of management approaches as well as types of documents through which management is conducted underscore the complexity and breadth of resource management, which must integrate multiple strategies and adapt to both changing ecological conditions and varying governance frameworks.

The U.S. Gulf of Mexico, which produces the greatest number of wild harvested native oysters in the world (Beck et al. 2011), has experienced extensive geographic and economic losses (Camp et al. 2015; Gledhill et al. 2020; Hesterberg et al. 2020). Gulf-wide, an estimated 4–8 billion subtidal oysters—240–508 million pounds of fresh oyster meat—were lost due to the 2010 Deepwater Horizon oil disaster alone (DWH NRDA 2017). Despite the common challenges facing oyster populations at the regional level (VanderKooy 2012), oyster fisheries in the U.S. Gulf of Mexico are managed by five different states (e.g., Texas, Louisiana, Mississippi, Alabama, Florida), each by a regulatory agency responsible for overseeing stock assessments, setting harvest limits, establishing size requirements, sustaining populations, and defining regulations and policies following state-specific management guidelines (Leard et al. 1999). Management decisions are informed by state-specific fishery management plans and other guidance documents that comprise both broad frameworks (e.g. management goals, objectives), and specific directives (e.g. monitoring and enforcement) to guide sustainable use. However, little is known about the specific content of these management documents or how their differences reflect state-level conditions and priorities that may affect oyster resources at the regional level. Understanding differences in oyster resource management strategies across political boundaries can help inform successful regional management of oysters, for example, by coordinating management decisions relative to large-scale environmental drivers (Kennish 2022; Tracy et al. 2023). Corpus linguistic techniques present an empirical opportunity to explore differences among those documents to inform coordinated management planning.

Corpus linguistics is an important, well-established subfield of the discipline of applied linguistics that collects and investigates large bodies of naturally occurring language quantitatively and qualitatively through computationally generated interfaces to identify and interpret linguistic patterns at scale (Biber and Reppen 2015; Cortes and Csomay 2015; Hunston 2022). A corpus is a large body of naturally occurring language data, usually, but not always, made up of multiple texts. Like all empirical methods, decisions about data sampling and analytical design must align with theoretical knowledge of the data type, research questions, and best practices for valid results. Corpus and computational linguistics methods are increasingly used to investigate linguistic data relevant to scientific concerns (e.g., Clarke 2024; Fitzsimmons-Doolan and Beseres Pollack 2023; Roy et al. 2022).

Keyword analysis is a corpus linguistics technique that identifies *keywords*, words that are “statistically characteristic of a text or set of texts” (Culpeper and Demmen 2015, p. 90). It does so by comparing a wordlist (lists of each lexical item—i.e., word—and its frequency count) from a corpus of interest to a wordlist from a larger general/reference corpus using log-likelihood statistics. This reveals unexpectedly frequent lexical items in the corpus of interest. These items are labeled *keywords*. The keywords indicate what makes the corpus of interest distinct compared to the reference corpus—often revealing what the corpus of interest is “about” (Jaworska and Themistocleous, 2018). What the keywords reveal about the corpus of interest is highly dependent upon the relationship between the corpus of interest and the reference corpus. In addition to aboutness, keywords can reveal register, temporal, content, or ideological differences, as well as any combination of the above (Culpeper and Demmen 2015). Once the keywords are identified, qualitative analysis of their use in the corpus of interest is necessary to interpret their significance in the corpus. For example, Bevitori (2015) used keyword analysis among other techniques to understand how the environment is presented in American presidential speeches over time. Her analysis identified *energy* as a keyword in a corpus of President Carter’s speeches and interpretation revealed it used in a discourse of conservation. When multiple corpora of interest are compared to one another (i.e., the reference corpus is another corpus of interest or an aggregation of corpora of interest), the resulting keywords reflect what makes each corpus of interest most distinct from the other corpora of interest (Gillings et al. 2023). The current study uses this design.

The application of corpus linguistic methods to a marine ecology management problem presented in this study constitutes a novel interdisciplinary approach. It has long been acknowledged that interdisciplinary methods are critical for addressing complex, pressing ecological problems such as management of natural resources (Bennett et al. 2016; Bennett et al. 2017; Campbell 2005; Ewel 2001). Collaborative problem framing, integration of theoretical frameworks, and use of knowledge and skills from multiple disciplines were all applied in this study in accordance with best practices in interdisciplinarity (Aboelela et al. 2007; Defila and di Giulio 2017). By applying corpus linguistic techniques to analyze oyster management guidance documents, this study offers new insights into the language and framing of resource management strategies. The result is a study which treats documents produced to inform oyster management decisions as data for systematic linguistic analysis.

Using corpus linguistics techniques, including keyword and concordance analyses, this study examines the most current oyster management guidance document for each U.S. Gulf state. The aims are to identify what makes each document distinct and to reveal unique emphases as well as opportunities for shared framing across the states. Because our research question focuses on how oyster management is articulated in state guidance, we analyzed the most recent available documents, which vary in purpose and publication date among states. This approach ensures a representative comparison of current management perspectives across the region. Although focused on oyster fisheries in the U.S. Gulf of Mexico, this study provides a methodological approach that can be adapted to inform natural resource management strategies in different contexts and other regions globally.

## Methods

For this study, the five most recent state guidance documents for oysters (Texas: Quast et al. 1988; Louisiana: Banks et al. 2016; Mississippi: Mississippi Department of Marine Resources 2021; Alabama: Dalrymple et al. 2021; Florida: Radabaugh et al. 2019) were identified and prepared for analysis. These documents vary in purpose and publication date, ranging from traditional fisheries management plans (e.g., Louisiana) to mapping and monitoring reports (e.g., Florida), reflecting the unique ecological, regulatory, and management contexts of each state. Despite these differences, they represent the most current written guidance available from each state at the time of analysis. Although the Texas Oyster Fishery Management Plan (Quast et al. 1988) is older than the other documents included in this study, it remains the only comprehensive state-issued guidance for oyster management in Texas. Despite regulatory updates over time, many management principles outlined in the plan continue to influence current policies and decision making, making it a relevant source for understanding the state’s approach to oyster resource management. Comparing these documents provides insight into the range of management approaches across the five Gulf states, from formal fisheries frameworks to adaptive management strategies and collaborative conservation initiatives. This variation highlights the broader continuum of management activities, including restoration efforts, conservation strategies, and ongoing monitoring. Understanding these differences is essential for examining how state-specific strategies are articulated and how they guide resource management in practice. Since this study aims to inform a real-world problem, analyzing the most current written guidance across states offers a practical framework for assessing oyster management practices.

Five keyword analyses were run in WordSmith (Scott 2016) with each document used as a corpus of interest and the other four documents together used as the reference corpus (p.01, log ratio 1.5) for each analysis. Thus, the keywords for each guidance document revealed what differentiated each text from the other four. The top keywords—those most unexpectedly frequent with respect to the other documents as measured by their log ratio score—were selected for further qualitative analysis. A log ratio score (Hardie 2014) is a measure of how unexpectedly common a particular keyword in a corpus of interest is. In other words, a log ratio score can be understood as an effect size measure for keywords and the top keywords are those that have the largest effect sizes (Gabrielatos 2018).

**Fig. 1:**
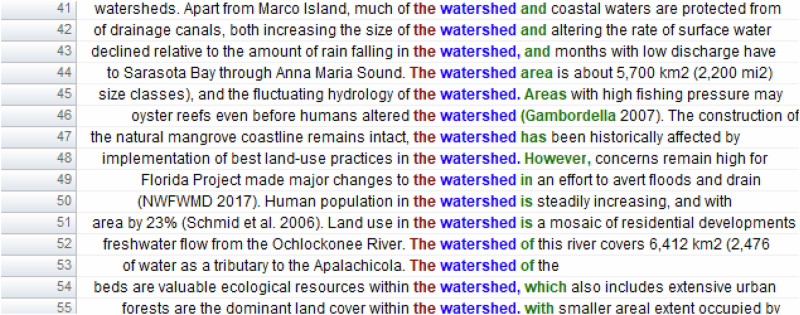
Concordance lines showing the top content keyword, *watershed*, in use in the Florida guidance document

Following the identification of top keywords for each guidance document, concordances (i.e., word occurrences in context; Fig. 1) and full co-text for each top keyword were examined by Author 1 to understand the context of use. Based on that analysis, the top keywords were placed into one of the following categories: number referring to numerical value (e.g., *76.301*), place (e.g., *Apalachee*), citation component (e.g., *accessed*), URL component (e.g., *geodata*), acronym (e.g., *ADCNR*) or content word (e.g., *adapt, proclamation, scraper*).

In the final round of analysis, for each state document, concordance lines as well as selected full co-texts for each *top content keyword* (*n* = 195 in total) were examined by Author 1 to create annotations with counts and descriptions of grammatical, thematic, and prosodic patterns of how each word was used in the document. Collocates (i.e. words that frequently co-occur with the keyword) were also included in the annotations. The resulting annotations were reviewed by Author 2 who added discipline specific observations. Next, for each state document (using these annotations), the top content keywords were grouped into themes by both authors together to better perceive and articulate top-level findings (Gillings et al. 2023). In addition, the ten most negative content keywords (i.e. statistically, unexpectedly *in*frequent content keywords in a particular guidance document compared the others; Culpeper and Demmen 2015) for each document were recorded. *Negative keywords*—the statistical opposite of keywords—indicate lexical items that are notably infrequent a given document. They are resulting lexical items from the statistical analysis that have negative values. We report the ten most negative (as measured by the log ratio score) content keywords for each document. The study results, therefore, include the following for each state oyster management guidance document: (1) total top keywords, (2) top keywords/word count, (3) the ten most negative content keywords, (4) top content keywords grouped by theme, and (5) results from the qualitative analysis of selected top content keywords which illustrate the top-level findings for each state.

## Results

The total word count for each individual U.S. Gulf state oyster management guidance document ranged from 8588 (Mississippi) to 90,093 (Florida) words. For the total top keywords (i.e. those with the largest effect sizes) Texas had the fewest (24) and Florida had the most (286). However, the ratio of top keywords to word count—a rough measure of how unique each plan is in relation to the others—shows that Louisiana and Alabama had the smallest ratios (0.0017 and 0.0018, respectively) indicating they are the least distinct, while Florida had the highest ratio (0.0032), indicating it is the most distinct relative to the others (Table 1).

**Table 1:** Total word counts, total top keywords, and ratio of top keywords to word count for each U.S. Gulf state oyster management guidance document

State plan	Total word count	Total top keywords	Top keyword/word count
Texas (1988)	9249	24	0.0026
Louisiana (2016)	83,532	139	0.0017
Mississippi (2021)	8588	26	0.0030
Alabama (2021)	28,363	52	0.0018
Florida (2019)	90,093	286	0.0032

### Texas

The 1985 Texas Legislature gave the Texas Parks and Wildlife Commission the authority to regulate the state oyster fishery through proclamation, pending the publication of a management plan. The Texas Oyster Fishery Management Plan (Quast et al. 1988) was published in 1988 by the Texas Parks and Wildlife Department to satisfy legislative requirements. The goal of the plan was to recommend strategies to optimize the yield and minimize depletion of the oyster fishery. The plan includes descriptions of eastern oyster life history, abundance trends, utilization patterns, and economic impacts of the Texas oyster fishery. After providing an overview of management structures over time, the plan makes recommendations such as “clarifying and simplifying existing regulations” (p. 38), area closures and time restrictions, and an overhaul of the lease system.

Of the ten most negative (unexpectedly *in*frequent) content keywords, *restoration* and *habitat* were the most negative (i.e. least represented; Table 2). The twelve top (most unexpectedly frequent) content keywords were grouped into seven themes (Table 3); three of these themes have only one top content keyword, suggesting they are underdeveloped. The remaining themes appear in other documents, although indicated by different keywords.

**Table 2:** Ten most negative content keywords in Texas’ oyster management guidance document

Most negative content keywords	Log ratio score
*restoration*	−5.13
*habitat*	−3.21
*quality*	−3.17
*cultch*	−3.09
*south*	−3.09
*substrate*	−3.08
*assessment*	−2.93
*new*	−2.87
*report*	−2.84
*crassostrea*	−2.74

**Table 3:** Top content keywords in Texas’ oyster guidance document grouped thematically

Theme	Top content keywords	Log ratio score
Top-down management	*proclamation*	138.41
	*overworked*	137.41
Values/quantitative	*coastwide*	139.53
	*ex (vessel)*	138.41
	*curve*	137.41
Spatial data/mapping	*unpolluted*	138.63
	*shaded*	137.41
Regulation of individual fishery participants	*fisherman’s*	137.41
Special activities	*stocking*	138.15
Biological processes	*hatch*	138.15
Monitoring	*minute*	137.41
	*min*	137.41

Among the top content keywords, *coastwide* and *overworked* were noteworthy (Table 4). *Coastwide* had the highest log ratio (139.53), occurred 13 times, was grouped into the theme *Values/quantitative*, and frequently co-occurred with *oyster, landings, abundance*. In 11 of the cases, *coastwide* was used as an adjective to modify nouns such as *abundance* (*n* = 3), *landings* (*n* = 4), and *total/s* (*n* = 2); in 10 of the 11 cases, the noun that it is modifying is being measured. Additionally, four uses of *coastwide* are associated with a top-down management decisions—particularly closures. *Overworked* only occurred three times. Yet, in each occurrence, overworked was used in conjunction with *damaged* to describe the condition of oyster reefs and to justify the management practice of harvest area closures.

**Table 4:** Selected top content keywords from Texas’ oyster guidance document in use

Exemplar No.	Top content keyword	Exemplar
1	*coastwide*	Seasonal closures should generally be applied **coastwide**
2	*overworked*	In five out of the last ten oyster seasons partial seasonal closures have been necessary because oyster populations were in a damaged or **overworked** condition either **coastwide** or in Galveston Bay

The bolded words in the exemplars are top content keywords for the state

### Louisiana

The Louisiana Oyster Fishery Management Plan was published by the Louisiana Department of Wildlife and Fisheries (Banks et al. 2016). The goal of the plan, which is subject to periodic revision, is to “assist with long-term conservation and sustainable use of the oyster resource for the maximum ecological, social, and economic benefit to the state of Louisiana” (p. 8). The plan consists of a description of the stock (including a biological profile and stock assessment), a description of the fishery (including quantitative landing data and other industry data), and a description of the ecosystem (including habitat and stressors). A description of the current management program is followed by a list of current challenges along with proposed solutions and research needs.

Among the ten most negative (unexpectedly infrequent) content keywords, *tidal*, *intertidal*, and *subtidal* stand out—suggesting that the Louisiana document does not differentiate between these reef types (Table 5). The 68 top (most unexpectedly frequent) content keywords were grouped into eight themes—the most of any of the five states (Table 6). *Regulation of individual fishery participants, Oysters as a product*, and *Law enforcement* are the most robust themes (i.e. highest number of content keywords), with *Oysters as a product* and *Dredging* occurring only in the Louisiana results.

**Table 5:** Ten most negative content keywords in Louisiana’s oyster management guidance document

Most negative content keywords	Log ratio score
*tidal*	−5.95
*intertidal*	−5.71
*islands*	−5.36
*district*	−5.18
*documents*	−4.99
*subtidal*	−4.95
*cover*	−4.72
*chapter*	−4.69
*parks*	−4.65
*priority*	−4.62

**Table 6:** Top content keywords in Louisiana’s oyster management guidance document grouped thematically

Theme	Top content keywords	Log ratio score
Regulation of individual fishery participants	*his*	137.35
	*nonresident*	137.23
	*valid*	137.23
	*possess*	136.65
	*calendar*	136.35
	*lessee*	136.35
	*anyone*	136.35
	*he*	136.35
	*refrigeration*	136.23
	*leaseholder*	136.23
	*owner*	136.23
	*lessees*	136.11
	*nonresidents*	135.82
	*submit*	135.82
Oysters as a product	*imports*	137.11
	*containers*	136.82
	*shucked*	136.82
	*frozen*	136.74
	*promotion*	136.46
	*salted*	136.35
	*dried*	136.35
	*tag*	136.11
	*untagged*	135.82
	*packaging*	135.82
	*official*	135.82
	*farmed*	135.82
	*brined*	135.82
	*growers*	135.65
Law enforcement	*violator*	137.60
	*offense*	137.35
	*imprisonment*	137.11
	*caught*	136.65
	*seized*	136.35
	*possessing*	136.35
	*taxes*	136.11
	*conviction*	135.97
	*forfeiture*	135.97
	*anything*	135.82
	*violations*	135.82
	*violators*	135.65
Top-down management	*parish*	137.29
	*authorities*	137.17
	*diversion*	136.56
	*legislation*	136.46
	*names*	136.35
	*moratorium*	136.11
	*endangered*	135.97
	*severance*	135.82
	*citizens*	135.65
	*exploration*	135.65
Monitoring	*sack*	139.34
	*biologists*	137.56
	*boarding*	137.17
	*bbls*	136.82
	*diversion*	136.56
	*bycatch*	136.23
	*replicates*	136.11
	*threshold*	136.11
	*week*	135.65
Special activities	*bedding*	137.90
	*reservations*	137.23
	*designate*	135.82
	*propagating*	135.65
Dredging	*scraper*	137.60
	*scrapers*	137.35
	*tooth*	136.35
Oyster stressor (competitor)	*fouling*	136.56
	*recurvum*	136.35
	*ischadium*	135.82

The top content keywords *sack*, *imports*, *scraper* and *violator* best illustrate the lexical distinctness of the Louisiana document (Table 7). *Sack* had the highest log ratio (139.34), was grouped into the theme of *Monitoring*, and collocated most frequently with *oysters, seed, barrels, oyster, stock*, and *limit*. *Sack* occurred 103 times in the plan, most often as an adjective: *sack oyster/s* (*n* = 33), *sack limit/s* (*n* = 11), *sack size/d* (*n* = 10), *sack stock* (*n* = 9). In these senses, *sack* refers to an oyster size that is harvestable as well as a management tool for limiting harvest. Many of the occurrences of *sack* were associated with monitoring efforts. *Sack limit* indicated a management tool for controlling the harvest and was also incorporated into descriptions of monitoring practices as an indicator of stock depletion. *Imports* occurred 22 times in the document and had the highest log ratio (137.11) in the theme, *Oyster as a product*, which was unique to Louisiana. In 18 of the occurrences, *imports* were being quantified either by amount or value and, in many of the cases, *imports* was followed by, “of farmed and wild live, fresh, frozen, dried, salted, or brine oysters.” *Imports* represents oyster imports to the U.S. and was primarily used to describe Louisiana’s commercial fishery as context for the state oyster industry. *Scraper*, used 31 times, had the highest log ratio (137.60) in the *Dredging* theme which was also unique to Louisiana. The most common collocates were *scraper, single, hand, oyster, nonresident, resident, licenses, tong, tongs, vessel, dredge, use, oysters, assist, limited, total*, and *mechanical*. It was used as an adjective in 13 cases (e.g., *scraper licenses, scraper teeth, scraper vessel*). The majority of the cases (*n* = 17) were associated with restricting the use of scrapers. Additionally, three cases explicitly acknowledge that *scraper* is the state-endorsed term for dredge. Lastly, *violator*, also used 31 times, had the third highest individual log ratio for the state (137.60) and was the highest in the *Law enforcement* theme, which was both robust and markedly punitive in the Louisiana results. In all occurrences, *violator* was used to describe the consequences for someone who had transgressed a statute.

**Table 7:** Selected top content keywords in use in Louisiana’s oyster management guidance document

Exemplar No.	Top content keyword	Exemplar
3	*sack*	August dredge samples found promising numbers of spat, seed and **sack** sized oysters at the western sites in the vicinity of Southwest Pass
4	*sack*	marked increase in the 2014 assessed **sack** stock coming behind three years of increases in seed oyster availability
5	*sack*	Through these interviews, they collect data on the number of sacks harvested per day and the time required to harvest the daily **sack** limit…LDWF considers harvest control measures if …substantial time is required to harvest the daily **sack** limit…
6	*imports*	Combined **imports** of live, fresh, **frozen,** **dried,** **salted**, and **brined** oysters from both **farmed** and **wild** sources, once a minority of imported oyster products, now make up the majority of oysters imported into the United States.
7	*scraper*	Commercial fishermen may only harvest oysters with tongs, a hand **scraper**, or a **scraper** with a **tooth** or flat bar no longer than 3 feet.
8	*scraper*	In 2016, legislation officially changed the name of dredge to **scraper**.
9	*violator*	the **violator** is prohibited from **possessing** an oyster harvester license
10	*violator*	the **violator** may not be present on a vessel harvesting or processing oysters.

The bolded words in the exemplars are top content keywords for the state

### Mississippi

The stated purpose of the Oyster Restoration and Recovery Plan (Mississippi Department of Marine Resources 2021) is to provide, “the current status of the commercial oyster industry in coastal Mississippi while simultaneously casting our vision to the future” (p. 1). It includes a short background section with an overview including the historical importance, environmental services, and recent decline of the oyster resource. Next, the goals and objectives for recovery of Mississippi oysters are briefly shared. The bulk of the document describes 30 funded and unfunded projects (e.g., restoration and aquaculture projects) designed to meet those goals.

Among the ten most negative (unexpectedly infrequent) content keywords, *river* and *freshwater* are notable given the challenges posed by freshwater flow to Mississippi oysters in recent years (Table 8). The 13 top (most unexpectedly frequent) content keywords were grouped into five themes with *Aquaculture* being unique to the state, the most robust (i.e. most keywords), and containing the keyword with the highest log ratio—*tidelands* (138.93; Table 9).

**Table 8:** Ten most negative content keywords in Mississippi’s oyster management guidance document

Most negative content keywords	Log Ratio Score
*river*	−4.08
*mapping*	−3.79
*2012*	−3.69
*license*	−3.23
*freshwater*	−3.22
*2014*	−3.10
*system*	−3.09
*between*	−3.00
*fishing*	−2.91
*fishery*	−2.78

**Table 9:** Top content keywords in Mississippi’s oyster management guidance document grouped thematically

Theme	Top content keywords	Log ratio score
Aquaculture	*tidelands*	138.93
	*efficiencies*	137.93
	*diploid*	137.93
	*borrowers*	137.52
	*loan*	137.52
Doc type: govt planning doc	*governor’s*	138.25
	*want*	137.93
	*unfunded*	137.52
Oyster stressor (human health)	*sewer*	137.93
	*septic*	137.52
Oyster restoration	*prime*	137.52
	*laden*	137.52
Link state to natural resources	*Mississippi’s*	137.52

Regarding the top content keywords in the Mississippi document, *tidelands* is used along with *efficiencies, diploids*, and other keywords to discuss *Aquaculture* (Table 10). Sixty-two percent of the occurrences of *tidelands* are used in relation to aquaculture activities. Similarly, all four cases of *efficiencies* described aspects of aquaculture projects or facilities supported by the state. All cases of *diploid* were also tied to aquaculture promotion. *Sewer* and *septic* in Mississippi’s plan were the only oyster stressors across the five Gulf states that were tied to human health. All cases (*n* = 4) of *sewer* and 2 of the 3 cases of *septic* occurred in a description of an unfunded project “Assist Coastal Municipalities Upgrade Faulty Sewer Systems,” designed to improve the environment for “Mississippi-grown oysters sold to the public” (p. 25).

**Table 10:** Selected top content keywords in use in Mississippi’s oyster management guidance document

Exemplar	Top content keyword	
11	*tidelands*	MDMR staff are committed to increasing the acreage at the Deer Island Oyster Aquaculture Park to 465 acres for Public Trust **Tidelands** Sub-leases and there are plans to develop aquaculture parks in each coastal county.
12	*efficiencies*	A demonstration facility will allow farmers to test new equipment, combine resources and streamline **efficiencies** so oyster farmers do not have to absorb the risk alone.
13	*efficiencies*	In partnership with the Mississippi Development Authority (MDA), the MDMR would like to provide affordable financing to oyster farmers and other parties who **want** to start or expand commercial shellfish aquaculture operations in Mississippi, but also **want** to ensure the sustainability of our public oyster reefs. Through this proposed program, the **borrowers** would agree to grow 15 percent of crops as **diploid** oysters that would be deployed on the wild reefs in Mississippi.
14	*sewer* *septic*	Unfortunately, through these surveys, the Shellfish scientists have documented many failing lift stations, overflowing manhole covers and **sewer** and **septic** leaks into the Mississippi Sound.

The bolded words in the exemplars are top content keywords for the state

### Alabama

Authored by the Alabama Department of Conservation and Natural Resources and the National Oceanic and Atmospheric Administration (NOAA), the Coastal Alabama Comprehensive Oyster Restoration Plan (Dalrymple et al. 2021) is a strategic document with the purpose to create and restore a resilient reef system “to support sustainable harvest and provide ecosystem services now and into the future” (p. iii). Current conditions for Alabama’s oyster resources are presented with attention placed on stressors. Historic and contemporary restoration activities are discussed in detail. Finally, the strategy is presented including prioritized activities with a focus on identifying sites, understanding relationships among sites, and adapting to environmental changes.

Among the ten most negative (unexpectedly infrequent) keywords were *license, retail, recreational*, and *season*, indicating that words relating to oyster harvest were statistically infrequent in this plan (Table 11). The 18 top (positive, most frequent) content keywords were organized into five themes, with *Oyster restoration* being the most robust (Table 12). *Patrol* had the highest log ratio score (140.30) not only among the Alabama top content keywords, but also among all the top content keywords across all five Gulf states.

**Table 11:** Ten most negative content keywords in Alabama’s oyster management guidance document

Most negative content keywords	Log ratio score
*2000*	−4.94
*grounds*	−4.44
*lagoon*	−4.38
*license*	−4.08
*season*	−3.90
*percent*	−3.82
*recreational*	−3.81
*recommendations*	−3.74
*retail*	−3.74
*pounds*	−3.68

**Table 12:** Top content keywords in Alabama’s oyster management guidance document grouped thematically

Theme	Top content keywords	Log ratio score
Oyster restoration	*configuration*	137.67
	*picture*	137.38
	*relic*	137.38
	*cured*	136.79
	*relayed*	136.53
	*configurations*	136.21
Law enforcement	*patrol*	140.30
	*you*	136.79
	*rating*	136.53
	*subtotal*	136.53
Doc type: strategy doc	*overarching*	137.53
	*exploring*	136.21
	*gain*	136.21
Top-down management	*adapt*	137.79
	*corrective*	137.38
	*planners*	137.02
Oyster stressors (acute)	*oiling*	137.02
	*Ivan*	136.21

Qualitative analysis of the top content keywords *patrol*, *configuration*, *oiling*, *Ivan* further clarified the lexical uniqueness of the Alabama document (Table 13). *Patrol* was used 68 times, primarily as an adjective (79% of the time), most often to describe *frequency, areas*, and *agency*. All cases, excepting two, occurred within the Marine Enforcement Protocol, presented in Appendix B of the document, that is used to evaluate patrol programs or as a description of shell stock patrol policies. *Patrol* and the other top content keywords grouped into the *Law enforcement* theme (i.e., *you, rating, subtotal*) present a proactive approach to law enforcement in contrast to Louisiana’s approach indexed by top content keywords such as *violator, offense*, and *imprisonment*, which was reactive. Used 11 times, *configuration* had the largest log ratio (137.67) of top content keywords grouped into the theme *Oyster restoration*. Its collocates were *reef, studies, oyster, cultch, relief*, and *section*. Uses of *configuration* either specified a particular project (*n* = 3) or named *configuration* as one of several variables affecting the efficacy of oyster restoration work (*n* = 8). While the *Oyster restoration* theme was not unique to Alabama, it only occurred in one other state—Mississippi, in which it is less developed. *Oiling* and *Ivan* were each used to discuss acute stressors to oysters. Uniquely, *oiling* was the only keyword to refer to damage to oyster reefs by oil leaks, whereas *Ivan* was the only keyword to refer to stress posed to oysters by hurricanes.

**Table 13:** Selected top content keywords in use in Alabama’s oyster management guidance document

Exemplar No.	Top content keyword	Exemplar
15	*patrol*	How am I going to assess whether the **patrol** agency has the resources (transportation, communication, and personnel) to meet required frequencies for each of the fifteen (15) **patrol** areas.
16	*patrol*	Are the vehicles suitable and adequate in number to achieve the minimum **patrol** frequency?
17	*configuration*	Reef **Configuration** Studies, will be useful to managers in determining the best design parameters for each selected site
18	*configuration*	**configuration** can affect the performance of constructed reefs
19	*oiling*	An estimated total of 8.3 million adult equivalent oysters were lost due to marsh **oiling** along Gulf Coast shorelines
20	*Ivan*	Hurricanes **Ivan** (Category 3, 2004) and Katrina (Category 3, 2005) devastated Alabama’s oyster reefs.

The bolded words in the exemplars are top content keywords for the state

### Florida

Florida’s Oyster Integrated Mapping and Monitoring Program Report (Radabaugh et al. 2019) is a highly collaborative document composed primarily of data-filled chapters written by different authors for each region of the state. The purpose of the document is to “identify the status of and management priorities for oysters and their habitats” (p. v). The executive summary and introduction chapters present the lifecycle of the eastern oyster, reef and harvesting descriptions, an overview of threats to oysters, and common monitoring and mapping practices. Following chapters focused on eight region-specific descriptions and recommendations, a conclusion chapter presents cross-cutting management, monitoring, and mapping recommendations including the recommendation to develop a “comprehensive fishery management plan” (p. 161).

The most negative (unexpectedly infrequent) content keywords include several that focus on human-related actions such as *harvesters, secretary*, and *equipment* (Table 14). The 81 top (most unexpectedly frequent) content keywords from the Florida guidance document grouped into seven themes of which *Spatial data/mapping, Habitats*, and *Oyster stressors* were the most robust (most keywords) and *Habitats* was unique to the state (Table 15).

**Table 14:** Ten most negative content keywords in Florida’s oyster management guidance document

Most negative content keywords	Log ratio score
*purposes*	−5.00
*secretary*	−4.90
*proposed*	−4.90
*feet*	−4.90
*culture*	−4.80
*stocks*	−4.76
*equipment*	−4.64
*achieve*	−4.64
*sized*	−4.64
*harvesters*	−4.52

**Table 15:** Top content keywords in Florida’s oyster management guidance document grouped thematically

Theme	Subtheme	Top content keywords	Log ratio score
Spatial data/Mapping		*fig*	139.68
		*ac*	138.52
		*ha*	138.37
		*photographs*	137.71
		*photography*	137.54
		*photo*	137.45
		*GIS*	137.35
		*credit*	137.18
		*color*	136.35
		*km*	136.35
		*topographic*	136.35
		*mi2*	136.24
		*km2*	136.24
		*geoform*	136.13
		*ft2*	136.13
		*mi*	136.13
		*graph*	136.00
		*shapefiles*	136.00
		*figs*	136.00
		*digital*	135.86
		*images*	135.71
		*features*	135.71
		*acoustic*	135.71
Habitats		*seagrass*	138.90
		*watershed*	138.32
		*roots*	137.90
		*seawalls*	137.63
		*creeks*	137.63
		*creek*	137.50
		*mangroves*	137.45
		*preserves*	137.45
		*urban*	137.30
		*hardened*	136.86
		*prop*	136.54
		*seagrasses*	136.35
		*inlets*	136.35
		*flats*	136.35
		*dominated*	136.35
		*fork*	136.13
		*root*	136.00
		*spoil*	135.86
		*mangle*	135.71
		*rhizophora*	135.71
		*seawall*	135.71
Oyster stressors	Intertidal	*wakes*	136.79
		*submergence*	135.86
		*linear*	135.71
	Threatening species	*boring*	136.54
		*Asian*	136.45
		*crown*	136.13
		*charruana*	136.00
		*barnacle*	136.00
		*mytella*	136.00
		*sponge*	136.00
		*conch*	135.86
		*nonnative*	135.86
	Water quality and quantity	*upstream*	136.63
		*extremes*	136.13
		*wet*	135.86
		*clarity*	135.71
		*downstream*	135.71
		*groundwater*	135.71
	*mining*		136.63
	*cope*		135.86
Values/Quantitative		*collapse*	137.86
		*tons*	136.63
		*1980s*	136.35
		*budgets*	136.24
		*die*	136.24
		*mercury*	135.86
Monitoring		*clumps*	136.24
		*horizontal*	136.13
		*manual*	135.86
		*subclass*	135.71
		*biota*	135.71
Doc type: Ad hoc, Bottom up		*download*	137.00
		*Ryan*	136.24
		*version*	135.71
		*chapters*	135.71
		*contacts*	135.71
Link state to natural resources		*Florida’s*	136.00

The results of the qualitative analysis for the top content keywords *fig*, *seagrass*, *watershed*, *collapse*, *crown, charruana*, and *mining* illustrate what sets the Florida document apart from the others (Table 16). The annotations of the top content keywords in the Florida document showed that *fig* had the highest log ratio score in the FL document and occurred 141 times, with the most common collocates of *bay, are, oyster, reefs, river*, and *rivers*. *Fig* stood for figures, which were often maps (*n* = 57) featuring waterbodies. In comparison, the Louisiana document contains (*n* = 19) maps. *Fig* was categorized in the theme *Spatial data/mapping*, which only occurred in one other state—Texas—and much less robustly. *Seagrass* (138.90) and *watershed* (138.32) had the highest log ratio scores among top content keywords grouped as *Habitats*. The most common collocates of *seagrass* were *oyster, mapping, beds, reefs, bay, SWFWMD, 2016, patchy, beaches, continuous, habitats*, and *extent*. The most common uses of *seagrass* were to present data related to seagrass and to describe habitats. The most common uses of *watershed* were as part of a proper noun, (e.g., “Naples Bay watershed”) and to suggest that or describe how people/development affect the watershed, usually negatively. Almost all 40 cases of *collapse* were related to the collapse of the Apalachicola 2012 oyster fishery—focusing on both causes as well as results. Its collocates were *oyster, fishery, bay, Apalachicola, 2012, 2013, 2015, population, 2012, since 2011, resulted, before, prior, historic, warning*, and *reef*. Lastly, though *Oyster stressors* was identified as a theme among the top content keywords in each state except Texas, it was especially robust in the Florida results, with 20 keywords subdivided into five subcategories of stressors. *Crown, charruana*, and *mining* were among the top content keywords in this category. In all cases, *crown* is used with *conch* to reference a predatory species (i.e., crown conch, *Melongena corona*) for oysters. Similarly, *charruana* is used with *mytella* to reference an invasive species (i.e., charru mussel, *Mytella charuanna*) that competes with oysters. In addition to stressing oyster reefs, the distribution of both species both lexically and geographically may point to tropicalization effects due to climate change (e.g., Vergés et al., 2014) which was not indicated in any other state’s results. *Mining* (collocates: *shell, construction, harvesting, bay, Tampa, dredging, glimpse*) was used in reference to shell mining and often (59% of cases) in discussion of habitat loss. It is the only top content keyword that points to a Gulf-wide historical practice which transformed the essential structure of oyster reefs across all five states (Doran 1965).

**Table 16:** Selected top content keywords in use in Florida’s oyster management guidance document

Exemplar No.	Top content keyword	Exemplar
21	*fig*	Choctawhatchee Bay (**Fig**. 2.6) receives freshwater flow.
22	*seagrass*	The data layers include **seagrass** persistence, aquaculture lease areas, boat channels, bathymetry, and tidal river isohalines
23	*seagrass*	**Mangroves dominated** the shoreline of this shallow estuary, and the bay once thrived with oyster reefs, **seagrass** beds, and numerous fish species
24	*watershed*	Land use in the **watershed** is a mosaic of residential developments, industrial areas, and agricultural lands, which have increased the pollutant load to the bay
25	*watershed*	Historically, these **creeks** drained a small **watershed**, but as the area was developed, many of the **creeks** were disconnected by a series of water management canals and mosquito control ditches.
26	*collapse*	The 2012 fishery **collapse** in the bay also resulted in a loss of some portion of the ecosystem services the oyster reefs provided
27	*collapse*	The **collapse** thus had ecological as well as economic and social effects
28	*crown*	this decline coincides with an increase in occurrence of the predatory **crown conch**
29	*mining*	A large portion of historical losses was due to activities that are now restricted by environmental regulations, such as dredge-and-fill construction and shell **mining** on live reefs

The bolded words in the exemplars are top content keywords for the state

## Discussion

This study used corpus linguistics techniques to analyze oyster management guidance documents from the five U.S. Gulf states, revealing how the texts were distinct through statistically identified keywords as well as shared framings among those distinctions that have implications for regional management. Florida’s document was the most distinctive, emphasizing issues like oyster stressors and habitat. Louisiana’s document reflected its role as a major oyster producer. Mississippi’s document uniquely emphasized aquaculture as a recovery strategy for environmental stressors. The theme of oyster restoration was robust in Alabama’s (2021) document, but absent the Texas (1988) document, indicating temporal differences in management foci. For example, the absence of explicit oyster restoration themes in the Texas document may reflect that management priorities at the time were more focused on immediate resource recovery following events such as Hurricane Elena (Berrigan 1988). Although each of the documents addresses state-specific ecological and political issues, there are also shared challenges that could inform regional management strategies (e.g. VanderKooy 2012). The study provides a methodological framework that is adaptable to other natural resource management contexts and other regions globally.

Grouping the top content keywords (e.g., *imports, diploid, configuration, mangroves*) into themes based on an examination of their use in context—demonstrated that each of the oyster management guidance documents, except for Texas’, had at least one thematic focus that distinguished it from the others. *Oysters as a product* was unique to Louisiana, and *Regulation of individual fishery participants* was far more robust in the Louisiana results than in Texas (where it was only represented by one top content keyword). These two themes may reflect Louisiana’s dominance as an oyster producer in the U.S. Gulf (Wirth and Minton 2004; NOAA 2024). *Aquaculture* was unique to the Mississippi document and was presented as a strategy for recovery in response to stressors such as diversions and storms (discussed in Pruett et al. 2024). *Oyster restoration* was also identified in Mississippi’s document, but it was much more explicit and robust in the Alabama plan. Although both states have a robust body of scientific literature focused on oyster restoration, the Mississippi approach was generally reactive and focused on recovery (e.g. Gledhill et al. 2020; Morgan and Rakocinski 2022) compared to Alabama’s more proactive focus on ecological enhancement (Gregalis et al. 2009; Scyphers et al. 2011). Finally, the theme of *Habitats* was unique to the Florida document, and while *Spatial data/mapping* was present in the Texas results, it was far more robust in the Florida document. While the Texas document had a word count and a keyword/word count ratio similar to that of the Mississippi document, the Texas top content keywords were distributed across more themes, such that only four themes have more than one keyword, none of which were distinct relative to the other documents.

Lexical differences in shared themes demonstrate the diversity of local conditions and management approaches, which is insightful in understanding state-specific challenges and practices. For example, the theme *Oyster stressors* was identified in results from all the states except Texas. However, several subcategories were identified: *competitors* (Louisiana)*, human health* (Mississippi)*, acute* (Alabama)*, intertidal, threatening species*, and *water quality and quantity* (Florida), indicating that while oysters are under stress (and pose management challenges) across the U.S. Gulf region, the types of stress differ by each state’s local conditions (see zu Ermgassen et al. 2012). Notably, Florida’s plan (2019)—which was written after the collapse of the Apalachicola Bay oyster fishery and federal fisheries disaster declaration of 2012, and just prior to the 5-year closure of the bay in 2020 (detailed in Brown et al. 2021)—included more *Oyster stressor* keywords and subthemes than any other document, and may reflect a specific stage in policy formulation. The theme of *Law enforcement* was also identified in multiple states (Louisiana, Alabama), however, differences among the top content keywords in each (e.g., *violator, offense, imprisonment*: LA*; patrol, you, rating*: AL) pointed towards distinct approaches to law enforcement (i.e., proactive in AL vs. reactive in LA). Finally, *Top-down management* was identified as a theme in the Texas (1988), Louisiana (2016), and Alabama (2021) results, but pointed towards different approaches. For example, *proclamation* and *overworked* in Texas were used to indicate a shift in management agent/process as well as an extreme status of the fishery associated with a management action (i.e., closures). Similarly, Louisiana’s top content keywords in this theme (e.g., *authorities, legislation, moratorium*) were used to refer to management agents, processes, and actions (sometimes extreme). In contrast, the *Top-down management* keywords in in the Alabama results (i.e., *adapt, corrective, planners*) were used to refer to the importance of resource managers and planners using ongoing environmental and social data collection to change management actions. The increasing emphasis on adaptive management approaches in recent documents aligns with recommendations for effective ecological conservation and management in dynamic environments (NRC 2017; Pine et al. 2022; Deepwater Horizon Natural Resource Damage Assessment Trustees 2017).

The distribution of the *Oyster restoration* keywords across states was noteworthy and reflected a growing emphasis on restoration efforts over time, as demonstrated by Bersoza Hernández et al. (2018). Oyster restoration was prominent in the Alabama (2021) results as indicated by the top content keywords: *configuration, picture, relic, cured, relayed, configurations*, and was also present in the Mississippi (2021) results, though much less robustly. Although *restoration* was discussed to some extent in the Louisiana (2016) and Florida (2019) plans, it was notably absent in the Texas (1988) results, and was the most negative keyword along with *habitat*, *cultch*, and *substrate*. The explicit and robust nature of this theme in the Alabama document is likely attributed to its emphasis as an “Oyster Strategy Document” (Dalrymple 2021, p.1) on restoration activities as a companion to the state’s 2016 Oyster Management Plan. That presence of multiple negative keywords related to restoration in the Texas results means that this theme was unexpectedly infrequent in the Texas document. This is surprising given that *overworked*, which co-occurred in the document with *damaged*, indicates an explicit awareness of the degraded condition of the state’s oyster resources. Since the Alabama and Mississippi documents were the most recent, while the Texas document is the oldest, the growing emphasis on oyster restoration in newer documents reflects a broader shift toward data-driven and modeling approaches to support restoration efforts (La Peyre et al. 2021; LDWF 2022). The shift toward emphasizing multiple ecological functions in oyster restoration is consistent with broader trends in the scientific literature highlighting the need to conserve oyster reef habitats for benefits beyond just fisheries production (Lenihan and Peterson 2004; Ruesink et al. 2005; Coen et al. 2007; Grabowski et al. 2012)—which may have influenced its increasing prominence within management documents.

Notable patterns within the findings indicate that state-based differences in terminology—not oyster management guidance—also drive differences observed among documents. For example, Louisiana’s theme *Dredging* was identified based on the top content keywords: *scraper, scrapers*; although the words ‘dredger’ and ‘scraper’ have historically been used in Louisiana to refer to the same harvesting device (Dymond 1904), the latter was legislated as the term for dredging in the state.

Future comparisons between state-level documents and regional management plans (VanderKooy 2012) could further highlight how management initiatives align or diverge across governance levels. These comparisons would reveal how management initiatives align or vary across levels, and highlight areas of consistency and/or divergence in oyster management practices across the U.S. Gulf. This methodological approach could also be applied to other natural resource management contexts with diverse management and geographic structures, both in terrestrial (e.g. cooperative forest management; FAO 2016) and marine (e.g. conservation planning across international boundaries; Kark et al. 2009) environments. Future research could also investigate which management approaches are most effective in achieving desired ecological results, for example, by examining the connection between specific practices emphasized within each document and measured ecological outcomes. Taken together, this approach can be used to inform and align management and policy strategies to support sustainable resource management across political boundaries and governance levels.

## Conclusions

This linguistic analysis of oyster management guidance documents from the five U.S. Gulf states reveals the distinctiveness of each plan, reflecting each state’s ecological and political contexts and offer insights for regional management. Florida’s guidance document focuses on oyster stressors and current management needs. Louisiana’s more traditional fishery management document reflects its dominance as an oyster producer in the Gulf. Mississippi’s document emphasizes the use of aquaculture and oyster restoration as recovery strategies, whereas the comprehensive focus of Alabama’s document on oyster restoration reflects a proactive approach to environmental challenges. The absence of certain themes in the oldest document—Texas’–points to a temporal evolution of management foci, for example an increasing emphasis on oyster restoration over time. This temporal shift is reflected in the scientific literature as well, where the rationale for oyster restoration has expanded from sustaining fisheries alone to supporting myriad ecosystem functions and services.

The linguistic differences identified in this study highlight the uniqueness of state-specific priorities and management approaches while also drawing attention to the need for greater integration at the regional level. While consideration of state-specific environmental conditions and challenges are necessary to the development of effective management strategies, this study identifies common themes across the differences (e.g. oyster stressors, oyster restoration) that provide a framework for advancing regional communication and collaborative management planning.

Finally, this study demonstrates an interdisciplinary methodological framework. The application of corpus linguistics techniques to evaluate natural resource management documents can be adapted to natural resource management plans across different geographic (e.g. terrestrial, marine) and spatial (e.g. state, regional, national, global) scales. In cases where natural resources cross political boundaries and are governed by multiple bodies, corpus linguistics analysis can help identify localized priorities, complementary terminology, and common challenges to facilitate more integrated management plans.

## Data Availability

The data generated and analyzed during this study, which include the guidance documents used for analysis and the statistical results of the keyword analyses, are available from the corresponding author upon reasonable request.
